# Changes in Physiological Parameters, Lipid Metabolism, and Expression of MicroRNAs in Genetically Improved Farmed Tilapia (*Oreochromis niloticus*) With Fatty Liver Induced by a High-Fat Diet

**DOI:** 10.3389/fphys.2018.01521

**Published:** 2018-10-30

**Authors:** Yi-Fan Tao, Jun Qiang, Jing-Wen Bao, De-Ju Chen, Guo-Jun Yin, Pao Xu, Hao-Jun Zhu

**Affiliations:** ^1^Wuxi Fisheries College, Nanjing Agricultural University, Wuxi, China; ^2^Key Laboratory of Freshwater Fisheries and Germplasm Resources Utilization, Ministry of Agriculture, Freshwater Fisheries Research Center, Chinese Academy of Fishery Sciences, Wuxi, China

**Keywords:** GIFT, lipid metabolism, miRNA, fatty liver, high-fat diet

## Abstract

Tilapia is susceptible to hepatic steatosis when grown in intensive farming systems. The aim of this study was to explore the mechanism of fatty liver induced by a high-fat diet (HFD) in genetically improved farmed tilapia (GIFT, *Oreochromis niloticus*). Juvenile GIFT were fed with HFD or a normal-fat diet (NFD) for 60 days. Substantial fat deposition in the liver of HFD-fed GIFT on days 20, 40, and 60 was observed using hematoxylin – eosin staining and oil red O staining. The increased fat deposition was consistent with increased triglyceride (TG) and total cholesterol (TC) levels in the liver of HFD-fed GIFT. There were significant differences (*P* < 0.05) in serum biochemical indexes (TG, TC, low density lipoprotein-cholesterol, and insulin contents, and alanine aminotransferase activity) between GIFT fed a HFD and GIFT fed a NFD on days 20, 40, and 60. Furthermore, 60 days of a HFD significantly changed (*P* < 0.05) the hepatic fatty acid composition, and led to increased polyunsaturated fatty acid levels and decreased saturated fatty acid and monounsaturated fatty acid levels. Hepatic antioxidant enzyme activities increased by day 20 and then declined, which led to an increase in malondialdehyde contents in the liver of HFD-fed GIFT. Molecular analyses revealed that the microRNAs miR-122, miR-29a, and miR-145-5p were upregulated, whereas miR-34a was downregulated in HFD-fed GIFT. *SCD*, *ELOVL6*, and *SRD5A2* encode three important enzymes in lipid metabolism, and were identified as potential targets of miRNAs. The transcript levels of hepatic *SCD* and *ELOVL6* were decreased and that of hepatic *SRD5A2* was increased in GIFT fed a HFD. Overall, the results of this study revealed a potential link between miRNAs and fatty liver induced by HFD, and suggest that a HFD could lead to excess fat deposition in the GIFT liver, which may disrupt hepatic lipid metabolism and reduce the antioxidant defense capacity.

## Introduction

Lipids are essential nutrients for fish. Dietary lipids at appropriate levels can provide energy and essential fatty acids that are beneficial for fish growth. Within certain limits, increasing dietary lipid levels can reduce the amount of protein required in the diet (Figueiredo-Silva et al., 2010; Zheng et al., 2010). However, a high-fat diet (HFD) can result in unwanted lipid accumulation in fish, resulting in fatty liver. Several studies have shown that fatty liver induced by a HFD is related to changes in blood biochemistry parameters, liver histology, and fatty acid composition. Such changes, which have been observed in blunt snout bream (*Megalobrama amblycephala*) (Lu et al., 2013a) and grass carp (*Ctenopharyngodon idella*) (Du et al., 2006), are indicators of abnormal metabolism and physiological status. Excess lipid intake has been shown to cause oxidative stress and affect the immune responses and disease resistance of marbled rockfish (*Sebastiscus marmoratus*) (Shi et al., 2013), turbot (*Scophthalmus maximus*) (Jia et al., 2017), and blunt snout bream (Adjoumani et al., 2017). Several studies have explored the mechanism of fatty liver formation in fish induced by a HFD (Lu et al., 2013b; Wang et al., 2015). The results of those studies suggested that hepatic steatosis induced by a HFD is strongly associated with abnormal lipid metabolism, including changes in lipid synthesis, uptake, and transport. For example, Lu et al. (2013b) showed that a HFD activated the activities of lipoprotein lipase and changed the mRNA expression levels of *PPAR*α, encoding peroxisome proliferator-activated receptor-α, and *PPAR*γ, encoding peroxisome proliferator-activated receptor-γ. Those changes resulted in the inhibition of lipid transport out of the liver and elevated lipid uptake, which contributed to fatty liver in blunt snout bream. However, few studies have focused on the physiology and molecular biology of fat deposition in tilapia.

With the rapid development of intensive fish farming (Malik et al., 2018), tilapia has become susceptible to hepatic steatosis when fed a HFD with an aim to use less protein (Ng and Romano, 2013; Huang et al., 2016, 2018). Several reports have shown that HFD-induced fatty liver is often accompanied by low growth and low immune function in farmed fish (Lu et al., 2013a; Ma et al., 2018). Therefore, it is particularly important to treat or alleviate HFD-induced fatty liver in tilapia. Genetically improved farmed tilapia (GIFT, *Oreochromis niloticus*) is bred from selected tilapia breeding stocks from Africa and Asia (Tendencia et al., 2006). Because of its excellent growth performance and disease resistance, GIFT is highly marketable and potentially has a broad market (Ng and Romano, 2013; Qiang et al., 2013). It is one of the most important commercial fish, and accounts for almost 75% of the total aquacultured tilapia in China at present. Thus, research to better understand the mechanism underlying the development of fatty liver in GIFT is particularly important from both biological and economic perspectives.

Recently, microRNAs (miRNAs) have emerged as key regulators of gene expression at the posttranscriptional level. These molecules are involved in multiple biological processes (Bartel, 2004). Thus, exploring the role of miRNAs can help us to better understand the underlying mechanisms that guide the physiological changes in living organisms. Most miRNAs in animals inhibit effective mRNA translation of target genes through imperfect complementary base-pairing with the 3′-untranslated region (3′-UTR) of their target mRNAs (Guo et al., 2010; Bizuayehu and Babiak, 2014). Many studies have revealed that miRNAs are important regulators of liver function in teleost fish (Her et al., 2011; Mennigen et al., 2014; Zhang et al., 2014; Wang et al., 2016). Particularly, miR-122 has been shown as a biomarker of liver injury (Florczyk et al., 2016) and play a role in hepatic lipid metabolism in grass carp (Wang et al., 2016), rainbow trout (*Oncorhynchus mykiss*) (Mennigen et al., 2014), and zebrafish (*Danio rerio*) (Her et al., 2011). Analyses of our deep sequencing data revealed that some miRNAs were differentially expressed in the liver between GIFT fed a HFD and those fed a normal-fat diet (NFD) for 60 days. This finding suggested that these miRNAs may play important roles in the GIFT liver in response to a high fat intake (Tao et al., 2017). In this study, we chose three clearly differentially expressed miRNAs (miR-29a, miR-145-5p, and miR-34a) and miR-122, a potential hepatic lipid metabolism-related miRNA in teleost fish (Her et al., 2011; Mennigen et al., 2014; Wang et al., 2016), to further explore their roles in hepatic lipid metabolism in GIFT.

In our previous study, miRanda software^1^ was used to predict potential target genes with miRNA target sites in their 3′-UTRs based on Nile tilapia whole genome sequence data^2^. The miRanda parameters and cutoffs were as follows: score ≥140 and free energy ≤-7 kcal/mol (Betel et al., 2010). Gene Ontology (GO)^3^ was used to detect the functions of the miRNAs’ target genes. The results of those analyses indicated that *SCD* (encoding stearoyl-coenzyme A desaturase), with the relatively high score (153) and low free energy (-25.0 kcal/mol) among target genes, may be a target of miR-122. The *SCD* gene also had a relatively high score (153) and low free energy (-16.07 kcal/mol) among potential target genes of miR-29a. The gene *ELOVL6*, which encodes elongation of the very long chain fatty acid protein 6, had a relatively high score (162) and low free energy (-23.17 kcal/mol) among target genes of miR-145-5p. The *SRD5A2* gene, which encodes steroid 5 alpha-reductase 2, had a relatively high score (167) and low free energy (-26.47 kcal/mol) among potential target genes of miR-34a (Tao et al., 2017).

Stearoyl-coenzyme A desaturase (SCD) is a rate-limiting enzyme in monounsaturated fatty acids (MUFA) synthesis, and catalyzes the conversion of palmitic acid (C16:0) and stearic acid (C18:0) into palmitoleic acid (C16:1) and oleic acid (C18:1), respectively (Heinemann and Ozols, 2003). ELOVL6 plays a crucial role in elongating saturated fatty acids (SFA) and MUFA with 12, 14, and 16 carbons to form 18-carbon fatty acids (Moon et al., 2001). SRD5A2 was shown to suppress lipogenesis by inhibiting the effects of cortisol (Nasiri et al., 2015). Because these three enzymes are important regulators in lipid metabolism, we selected their encoding genes as potential miRNA targets that warranted further analysis.

The principal goal of this research was to characterize the potential mechanisms of GIFT fatty liver formation by investigating changes in physiological indexes, miRNA expression levels, and transcript levels of potential lipid metabolism-related target genes in the liver of GIFT in response to a HFD. Our results suggest that further research on techniques to attenuate hepatic steatosis induced by HFD in GIFT is warranted.

## Materials and Methods

### Ethics Approval

The experimental protocols were approved by the Institutional Animal Care and Use Committee of Nanjing Agricultural University (Nanjing, China). The experiments were performed according to the Guide for the Care and Use of Laboratory Animals in China.

### Experimental Diets

A previous nutritional study on GIFT juveniles (Wang et al., 2011) specified that 7.67–9.34% dietary lipid levels were optimal for GIFT. Other studies have shown that dietary lipid level ≥15% could be used to construct a fatty liver model of tilapia (Huang et al., 2016; Ma et al., 2018). Incorporated to our previous research (Qiang et al., 2017a), we considered that 18.5% dietary lipid level was suitable to construct a HFD-induced fatty liver GIFT model. Therefore, in this study, we established diets with 8 and 18.5% lipids as the NFD and HFD, respectively. The composition and ingredients of these experimental diets are shown in Table 1. All ingredients were mixed, an appropriate volume of water was added, and then the mixture was pressed to form 1.5-mm granular wet pellets. The pellets were dried at room temperature for 72 h and stored at -20°C until use.

**Table 1:** Ingredients and composition of normal-fat diet (NFD) and high-fat diet (HFD).

Dietary lipid level (g/Kg dry diet)		

	NFD	HFD
Fish meal^∗)^	100.0	100.0
Casein	76.0	76.0
Gelatin	19.0	19.0
Corn starch	237.7	4.8
Soybean oil	68.0	170.0
Soybean meal	120.0	120.0
Cottonseed meal	150.0	150.0
Rapeseed meal	150.0	150.0
Vitamin premix^‡)^	5.0	5.0
Mineral premix^§^^)^	5.0	5.0
Choline chloride	5.0	5.0
Vitamin C phosphate ester	2.0	2.0
Ca(H_2_PO_4_)_2_	15.0	15.0
α-cellulose	47.3	178.2
Total	1000.0	1000.0
Crude protein	326.8	328.7
Crude lipid	80.7	184.1
Gross energy (KJ/g diet)	147.7	150.5


*^∗^American Seafood, purchased from Coland Feed Co., Ltd., Wuhan, P. R. China. Chemical composition: moisture: 4.26%; crude protein: 68.97% of dry matter; crude lipid: 8.97%; ash: 12.15%. **^‡^**Vitamin premix (mg/kg dry diet):V_*A*_ 10, V_*D*_ 0.05, V_*E*_ 400, V_*K*_ 40, V_*B1*_ 50, V_*B2*_ 200, V_*B3*_ 500, V_*B6*_ 50, V_*B7*_ 5, V_*B11*_ 15, V_*B12*_ 0.1, V_*C*_1000, inositol 2000, choline 5000. ^§^ Mineral premix (mg/kg dry diet): FeSO_*4*_⋅7H_*2*_O 372, CuSO_*4*_⋅5H_*2*_O 25, ZnSO_*4*_⋅7H_*2*_O 120, MnSO_*4*_⋅H_*2*_O 5, MgSO_*4*_ 2475, NaCl 1875, KH_*2*_PO_*4*_ 1000, Ca (H_*2*_ PO_*4*_)_*2*_ 2500.*

### Fish Rearing and Sample Collection

Healthy juvenile GIFT were chosen from Yixing Tilapia Breeding Base of the Freshwater Fisheries Research Center, Chinese Academy of Fishery Sciences. The fish were acclimatized in indoor plastic tanks and fed with commercial feed for 1 week. After acclimation, juvenile GIFT with an average initial body weight (BW) of 2.94 ± 0.03 g were randomly divided into two dietary groups with three tanks (0.8 m × 1 m diameter × height) per group (stocking density, 30 fish per tank). During the entire rearing period, the fish were kept in an aerated, flow-through system under a natural photoperiod. The water temperature was maintained at 28 ± 1°C and one-third of the water was changed every 3 days. The fish were hand-fed to apparent satiation twice a day (8:00 and 16:00). The feeding rate was calculated using the following formula: feeding rate (FR, %BW/d) = 100 × feed intake/[(final weight + initial weight)/2]/rearing days (Huang et al., 2012). No significant difference (*P* > 0.05) in FR was found between the HFD (3.32 ± 0.10) and NFD (3.52 ± 0.08) groups during the feeding period.

Prior to sampling, food was withheld from the fish for 1 day to reduce the effects of food intake on the physiological and biochemical indicators. Three fish per tank were randomly caught and anesthetized using MS-222 (100 mg/L; Argent Chemical Laboratories, Redmond, WA, United States) on days 20, 40, and 60. Blood samples were taken from the caudal vein and immediately centrifuged using the method described by Ma et al. (2015). The serum was collected and stored at -40°C until further analysis. At the same time, liver tissues collected from the sampled fish were frozen in liquid nitrogen, and then stored at -80°C until analyses of enzyme activities and mRNA levels. Another fish from each of the six tanks was dissected to collect liver tissue for histological analyses. At the end of the trial, liver tissues were collected from three fish per tank and stored at -40°C until fatty acid composition analysis.

### Blood Biochemical Analysis

We used a fully automatic biochemical analyzer (bs-400, MINDRAY, Shenzhen, China) to measure serum glucose, triglyceride (TG), total cholesterol (TC), high density lipoprotein-cholesterol (HDL-C), and low density lipoprotein-cholesterol (LDL-C) contents, and alanine aminotransferase (ALT) and aspartate transaminase (AST) activities in the serum samples. Reagents and test kits were purchased from MINDRAY. The serum insulin level was determined by radioimmunoassay using guinea pig anti-porcine-insulin antiserum as the antibody and ^125^I-labeled insulin as the tracer (Wu and Ho, 1987). This method was validated in tilapia by Lin et al. (1995).

### Histological Analyses

Liver samples collected from fish were washed with physiological saline and carefully divided into two parts. The first part was fixed with 4% paraformaldehyde for 24 h, dehydrated in a graded ethanol series, embedded in paraffin, and then cut into 4-mm sections using a paraffin microtome (Leica RM2235, Leica Microsystems, Wetzlar, Germany). The sections were stained with hematoxylin – eosin and observed under an optical microscope (E100, Nikon, Tokyo, Japan). The second part was flash-frozen in liquid nitrogen and then cut into 10-mm frozen sections using a freezing microtome (Leica 3050S). The sections were stained with oil red O solution for 15 min, rinsed with distilled water for 30 s, counterstained with Mayer’s hematoxylin for 3 min, and observed under an optical microscope. The reagents used for histological staining were purchased from the Nanjing Jiancheng Bioengineering Institute (Nanjing, China).

### Hepatic Lipid Index Assays

Total lipids were extracted from liver tissues according to Folch’s method (Folch et al., 1957) and hepatic TG and TC levels were measured using test kits purchased from the Nanjing Jiancheng Bioengineering Institute (Nanjing, China).

### Analysis of Hepatic Fatty Acids

Fatty lipids were analyzed as described previously (He J. et al., 2015). Briefly, all lipids were first extracted using a chloroform/methanol mixture (2:1) and then methylated in 1% sulfuric acid in methanol at 70°C for 3 h to generate fatty acid methylesters (FAMEs). The FAMEs were extracted in heptane and examined by gas chromatography using a GC-2010 instrument (Shimadzu, Kyoto, Japan). Fatty acids were identified by comparison with known standards (Sigma, St Louis, MO, United States) and quantified using the CLASS-GC10 GC workstation (Shimadzu).

### Analysis of Hepatic Enzyme Activity

Liver samples (about 0.1 g) were homogenized in precooled phosphate buffer (50 mmol L^-1^, pH 7.4) and then centrifuged for 20 min (4°C, 3000 *g*). The supernatant was used for determining hepatic enzyme activities. The malondialdehyde (MDA) concentration and the activities of superoxide dismutase (SOD) and glutathione peroxidase (GSH-Px) were measured as described by Zhang et al. (2008). Catalase (CAT) activity was measured as described by Aebi (1984). All assay kits were purchased from the Nanjing Jiancheng Bioengineering Institute (Nanjing, China).

### RNA Preparation and qRT-PCR Analysis

The changes in the relative expression levels of miRNAs and their potential target genes in the HFD and NFD groups on day 20, 40, and 60 were determined by quantitative real-time PCR analyses as described previously (Qiang et al., 2017b). Briefly, miRNAs were extracted using an miRNeasy kit (Takara, Otsu, Japan) and reverse-transcribed using the Mir-X^TM^ miRNA First-Strand Synthesis kit (Takara). The expression levels of miR-122, miR-34a, miR-145, and miR-29a were quantified using a Mir-X^TM^ miRNA qRT-PCR SYBR^®^ kit (Takara, Dalian, China) with the 7900HT Fast Real-Time PCR System (Applied Biosystems, Foster City, CA, United States). U6 sRNA was used as an internal control.

To analyze the transcript levels of potential miRNA target genes, total RNA was extracted using Trizol reagent (Invitrogen, Carlsbad, CA, United States) and reverse-transcribed using Prime Script^TM^ RT Master Mix (Takara). The transcript levels of *SCD*, *ELOVL6*, and *SRD5A2* were quantified using a SYBR^®^ Premix Ex Taq kit (Takara). β-*Actin* was used as the reference control. All primers were synthesized by Genecore Biotechnologies Co., Ltd. (Shanghai, China) (Tables 2, 3). Relative expression was analyzed using the 2^-ΔΔCt^ method. Values relative to those in the NFD group on day 20 represent *n*-fold difference.

**Table 2:** miRNA primer sequences.

miRNA name	miRNA sequence (5′ to 3′)	specific forward primer (5′ to 3′)
miR-145-5p	GUCCAGUUUUCCCAGGAAUCCC	GTCCAGTTTTCCCAGGAATC
miR-29a	UAGCACCAUUUGAAAUCGGUUA	GCACCATTTGAAATCGGTTAG
miR-34a	UGGCAGUGUCUUAGCUGGUUGU	TGGCAGTGTCTTAGCTGGTTGT
miR-122	UGGAGUGUGACAAUGGUGUUUG	CTGGAGTGTGACAATGGTGTTT


**Table 3:** Primers and GenBank accession numbers of predicted target genes of miRNAs.

Target mRNA	Sequence	GenBank accession no
ELOVL6	F: 5′- ACAGTTCAACGAGGACGAAGC -3′	XM_003443399.3
	R: 5′- AGCAAGGGTGAGTGACCACAG -3′	
SRD5A2	F: 5′- ATACTCACCACGCACAAATCCAC -3′	XM_003441028.3
	R: 5′- AAACCCGCTGCCACCATC -3′	
SCD	F: 5′- ACAAGCTCTCCGTGCTGGTCAT-3′	XM_003441797.4
	R: 5′- GCAGAGTTGGGACGAAGTAGGC -3′	
Control gene β-*actin*	F: 5′- CCACACAGTGCCCATCTACGA -3′	EU887951.1
	R: 5′- CCACGCTCTGTCAGGATCTTCA -3′	


*ELOVL6, Elongation of the very long chain fatty acid protein 6; SRD5A2, steroid 5 alpha-reductase 2; SCD, stearoyl-coenzyme A desaturase.*

### Statistical Analysis

All results shown in figures and tables are mean ± standard error. Each value is the average of nine replicates. Data were tested for normality and homogeneity of variance using the Shapiro–Wilk test and the Levene test, respectively. Then, differences between the HFD and NFD groups at each sampling time were detected using independent-sample *t* test. Differences in the same treatment among different sampling points were analyzed using one-way ANOVA with *post hoc* Duncan’s multiple range tests (Supplementary Table S1). A *P*-value of <0.05 was considered statistically significant. All analyses were conducted using SPSS ver. 22.0 (SPSS Inc., Chicago, IL, United States).

## Results

### Serum Biochemical Indexes

**FIGURE 1:**
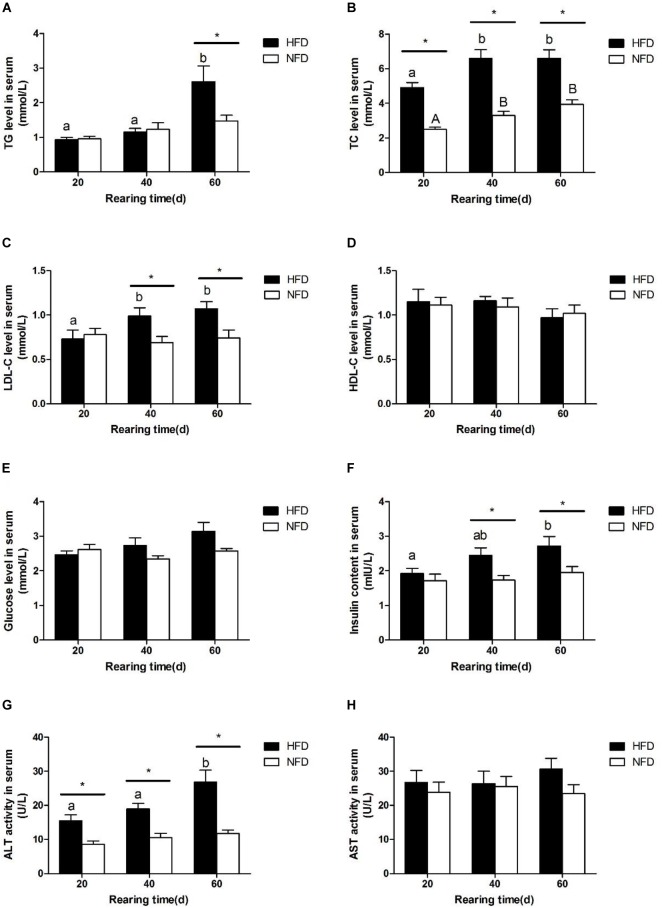
Serum biochemical parameters of GIFT fed experimental diets on day 20, 40, and 60 (*n* = 9 replicates per group). **(A)** Triglyceride, TG; **(B)** total cholesterol, TC; **(C)** low density lipoprotein-cholesterol, LDL-C; **(D)** high density lipoprotein-cholesterol, HDL-C; **(E)** glucose; **(F)** insulin; **(G)** alanine aminotransferase, ALT; **(H)** aspartate aminotransferase, AST. Significant differences (*P* < 0.05) between high-fat diet (HFD) and normal-fat diet (NFD) groups at same sampling time are marked by asterisks (^∗^). Different superscript lowercase letters show significant differences (*P* < 0.05) in same group among different sampling times. Different superscript uppercase letters show significant differences (*P* < 0.05) in NFD group among different sampling times.

We measured the serum biochemical indexes in GIFT fed a HFD and those fed a NFD on days 20, 40, and 60 (Figure 1). There was a significant increase (*P* < 0.05) in the serum TG level in the HFD group on day 60 (Figure 1A). The serum TC level (Figure 1B) and LDL-C level (Figure 1C) were significantly higher (*P* < 0.05) in the HFD group than in the NFD group on days 20 and 40, respectively. However, there was no significant difference (*P* > 0.05) in serum HDL-C contents (Figure 1D) between the HFD and NFD groups during the 60-day experiment. Compared with the NFD group, the HFD group showed an upward trend in serum glucose levels (Figure 1E), but the difference between these two groups was not significant (*P* > 0.05). The changes in the serum insulin content were similar to those in serum LDL-C levels, and a significant increase (*P* < 0.05) in serum insulin content was detected in the HFD group on days 40 and 60 (Figure 1F). The serum ALT activity was significantly higher (*P <* 0.05) in the HFG group than in the NFD group on days 40 and 60 (Figure 1G). The serum AST activity did not change in either group during the 60-day experiment (Figure 1H).

### Hepatic TG and TC Levels

**FIGURE 2:**
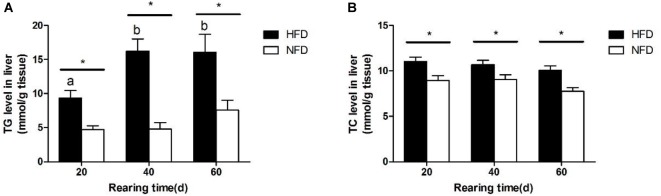
Levels of **(A)** triglyceride, TG and **(B)** total cholesterol, TC in liver of GIFT fed experimental diets on day 20, 40, and 60 (*n* = 9 replicates per group). Significant differences (*P* < 0.05) between high-fat diet (HFD) and normal-fat diet (NFD) groups at same sampling time are marked by asterisks (^∗^). Different superscript lowercase letters show significant differences (*P* < 0.05) in same group among different sampling times.

The changes in hepatic TG and TC levels during the 60-day experiment are shown in Figure 2. Both the hepatic TG (Figure 2A) and TC (Figure 2B) levels were significantly higher (*P <* 0.05) in the HFD group than in the NFD group after 20 days. Fish fed with the HFD showed a significantly higher (*P* < 0.05) hepatic TG level on days 40 and 60 than on day 20.

### Hepatic Antioxidant Capacity

**FIGURE 3:**
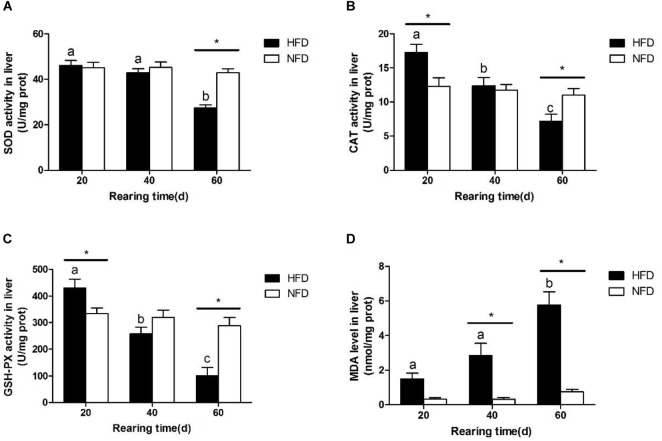
Oxidative stress status in liver of GIFT fed experimental diets on day 20, 40, and 60 (*n* = 9 replicates per group). **(A)** Superoxide dismutase, SOD; **(B)** catalase, CAT; **(C)** glutathione peroxidase, GSH-Px; **(D)** malondialdehyde, MDA. Significant differences (*P* < 0.05) between high-fat diet (HFD) and normal-fat diet (NFD) groups at same sampling time are marked by asterisks (^∗^). Different superscript lowercase letters show significant differences (*P* < 0.05) in HFD group among different sampling times.

Hepatic SOD activity (Figure 3A) was significantly lower in the HFD group than in the NFD group on day 60. During the 60-day experiment, hepatic CAT (Figure 3B) and GSH-Px (Figure 3C) activities first increased and then decreased in GIFT fed a HFD. Compared with the NFD group, the HFD group showed significantly higher (*P* < 0.05) hepatic CAT and GSH-Px activities on day 20, but significantly lower (*P* < 0.05) hepatic CAT and GSH-Px activities on day 60. The GIFT fed a HFD showed a gradual increase in MDA levels from day 20 to day 60. The hepatic MDA level (Figure 3D) was significantly higher (*P <* 0.05) in the HFD group than in the NFD group on day 40.

### Hepatic Fatty Acids Composition

As shown in Table 4, the hepatic fatty acids composition differed significantly between the HFD group and the NFD group on day 60. Compared with the NFD group, the HFD group should a significantly greater (*P <* 0.05) proportion of polyunsaturated fatty acids (PUFA) and a significantly smaller (*P <* 0.05) proportion of monounsaturated fatty acids (MUFA) and saturated fatty acids (SFA). Among the various PUFA, only n-6 PUFA showed a significantly higher (*P* < 0.05) concentration in the HFD group than in the NFD group. The n-3 PUFA content did not differ significantly (*P >* 0.05) between the two groups.

**Table 4:** Fatty acid composition in liver in GIFT fed a HFD or NFD for 60 days (% of total FAMEs).

	HFD	NFD
C12:0	0.02 ± 0.00*	0.07 ± 0.01
C14:0	0.84 ± 0.05*	2.81 ± 0.27
C15:0	0.12 ± 0.01*	0.06 ± 0.01
C16:0	14.83 ± 0.54*	26.98 ± 0.82
C17:0	0.30 ± 0.02*	0.13 ± 0.01
C18:0	7.26 ± 0.25*	14.90 ± 0.79
C20:0	0.17 ± 0.01	0.18 ± 0.01
C22:0	0.05 ± 0.01	0.06 ± 0.00
ΣSFA	23.57 ± 0.81*	45.18 ± 1.80
C16:1	1.47 ± 0.05*	2.90 ± 0.05
C18:1	29.57 ± 0.13*	32.76 ± 1.08
C20:1	0.79 ± 0.03*	1.05 ± 0.02
C22:1	0.59 ± 0.04*	0.04 ± 0.00
ΣMUFA	32.42 ± 0.19*	36.76 ± 1.14
C18:2n-6	35.16 ± 1.16*	10.39 ± 1.41
C18:3n-3	1.92 ± 0.17*	0.64 ± 0.12
C18:3n-6	0.76 ± 0.02*	0.41 ± 0.02
C20:2n-6	1.81 ± 0.07*	0.76 ± 0.12
C20:3n-3	0.79 ± 0.05	0.63 ± 0.09
C20:4n-6	1.24 ± 0.21	1.50 ± 0.31
C20:5n-3 (EPA)	0.22 ± 0.02*	0.05 ± 0.01
C22:3	0.30 ± 0.02	0.40 ± 0.08
C22:4n-6	0.31 ± 0.05	0.54 ± 0.13
C22:5n-3	0.23 ± 0.03	0.30 ± 0.07
C22:6n-3 (DHA)	1.29 ± 0.14	2.45 ± 0.56
ΣPUFA	44.01 ± 1.00*	18.06 ± 2.79
Σn-3PUFA	4.43 ± 0.13	4.07 ± 0.82
Σn-6PUFA	39.27 ± 0.92*	13.59 ± 1.91


*Data are mean ± SE, *n* = 9 replicates per group. Significant differences (*P* < 0.05) between high-fat diet (HFD) and normal-fat diet (NFD) groups are marked by asterisks (^∗^). SFA, saturated fatty acid; MUFA, monounsaturated fatty acid; PUFA, polyunsaturated fatty acid; EPA, eicosapentaenoic acid; DHA, docosahexaenoic acid.*

### Histological Structure of Liver

**FIGURE 4:**
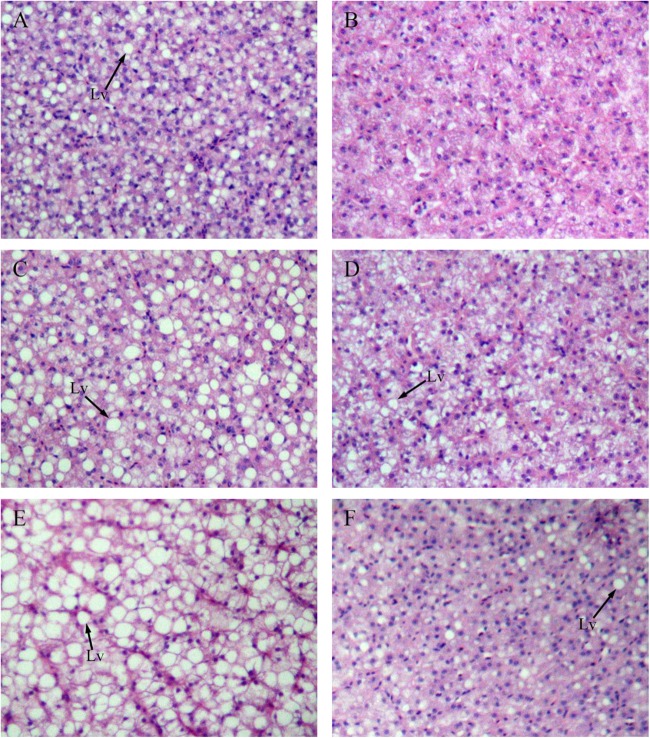
Hematoxylin-eosin staining of liver tissues of GIFT fed experimental diets on day 20, 40, and 60. Magnification 200×. **(A)** Fish fed high-fat diet (HFD, 18.5% lipid level) for 20 days; **(B)** fish fed-normal fat diet (NFD, 8% lipid level) for 20 days; **(C)** fish fed HFD for 40 days; **(D)** fish fed NFD for 40 days; **(E)** fish fed HFD for 60 days; **(F)** fish fed NFD for 60 days. Lv, lipid vacuole.

**FIGURE 5:**
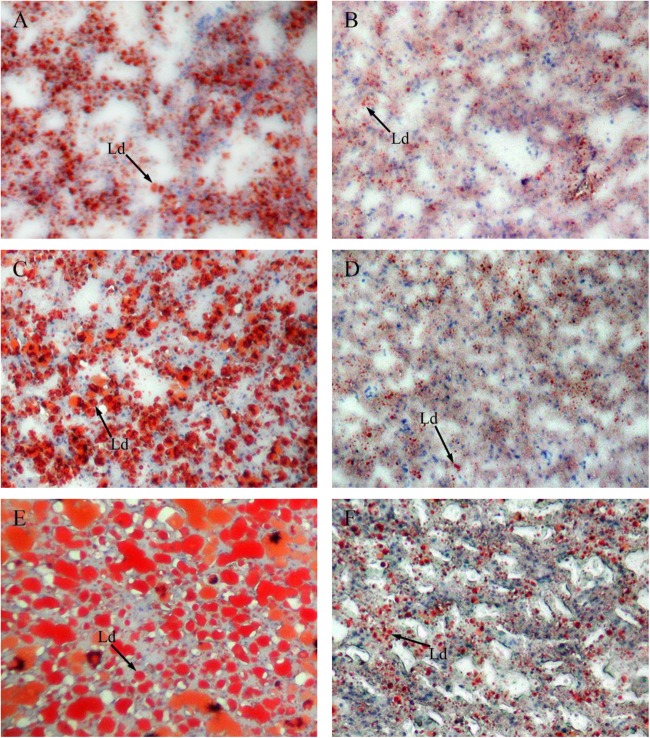
Red oil O staining of liver tissues of GIFT fed experimental diets on day 20, 40, and 60. Magnification 200 ×. **(A)** Fish fed high-fat diet (HFD, 18.5% lipid level) for 20 days; **(B)** fish fed normal-fat diet (NFD, 8% lipid level) for 20 days; **(C)** fish fed HFD for 40 days; **(D)** fish fed NFD for 40 days; **(E)** fish fed HFD for 60 days; **(F)** fish fed NFD for 60 days. Ld, lipid droplet.

Figure 4 shows sections of GIFT liver stained with hematoxylin and eosin. Compared with the liver of GIFT fed a NFD (Figures 4B,D,F), the liver of GIFT fed a HFD (Figures 4A,C,E) accumulated more and larger lipid vacuoles during the 60-day experiment. Oil red O staining was used to detect neutral lipids and lipid droplets in liver tissues of both HFD-fed GIFT (Figures 5A,C,E) and NFD-fed GIFT (Figures 5B,D,F). Oil red O-stained areas of lipid droplets occupied almost the entire area of liver sections from HFD-fed GIFT, but not NFD-fed GIFT, on day 60. These results indicated that severe hepatic lipid accumulation occurred in the HFD-fed GIFT.

### Expression of miRNAs in Liver

**FIGURE 6:**
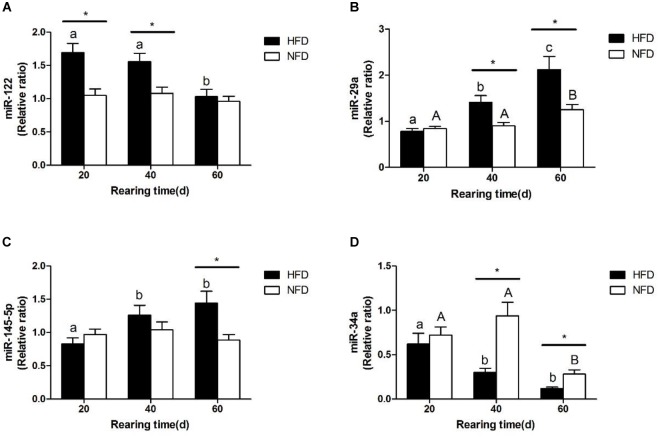
Expression levels of **(A)** miR-122; **(B)** miR-29a; **(C)** miR-145-5p and **(D)** miR-34a in liver of GIFT fed experimental diets on day 20, 40, and 60 (*n* = 9 replicates per group). Significant differences (*P* < 0.05) between high-fat diet (HFD) and normal-fat diet (NFD) groups at same sampling time are marked by asterisks (^∗^). Different superscript lowercase letters show significant differences (*P* < 0.05) in HFD group among different sampling times. Different superscript uppercase letters indicate significant differences (*P* < 0.05) in NFD group among different sampling times.

The changes in the expression levels of miR-122, miR-34a, miR-145-5p, and miR-29a are shown in Figure 6. The miR-122 expression levels (Figure 6A) were significantly higher (*P* < 0.05) in the HFD group than in the NFD group on days 20 and 40. The expression levels of miR-29a and miR-145-5p were significantly higher (*P* < 0.05) in the HFD group than in the NFD group on days 40 and 60 (Figures 6B,C). However, the miR-34a level was significantly lower (*P* < 0.05) in the HFD group than in the NFD group on days 40 and 60, and significantly lower (*P <* 0.05) in both the HFD and NFD groups on day 60 than on day 20 (Figure 6D).

### Expression of Lipid Metabolism-Related Genes in Liver

**FIGURE 7:**
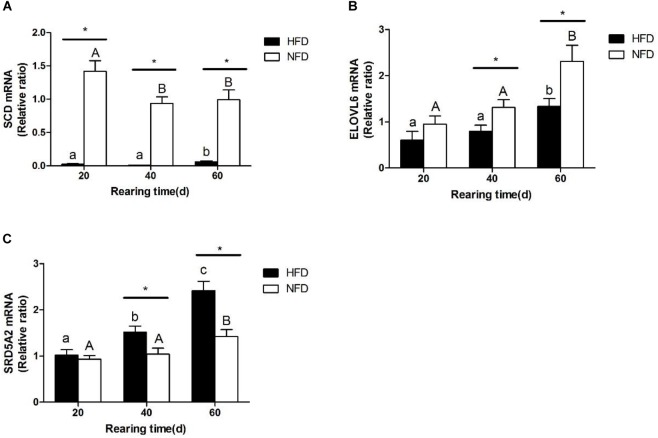
Expression levels of **(A)** *SCD* mRNA; **(B)** *ELOVL6* mRNA, and **(C)** *SRD5A2* mRNA in liver of GIFT fed experimental diets on days 20, 40, and 60 (*n* = 9 replicates per group). Significant differences (*P* < 0.05) between high-fat diet (HFD) and normal-fat diet (NFD) groups at same sampling time are marked by asterisks (^∗^). Different superscript lowercase letters show significant differences (*P* < 0.05) in HFD group among different sampling times. Different superscript uppercase letters show significant differences (*P* < 0.05) in NFD group among different sampling times.

The changes in the transcript levels of potential miRNA target genes (*SCD*, *ELOVL6*, *SRD5A2*) are shown in Figure 7. The transcript level of *SCD* was significantly lower (*P* < 0.05) in the HFD group than in the NFD group on day 20 (Figure 7A). The transcript level of *ELOVL6* was significantly lower (*P* < 0.05) in the HFD group than in the NFD group on days 40 and 60, and it was significantly higher (*P <* 0.05) in both the HFD and NFD groups on day 60 than on day 20 (Figure 7B). The transcript level of *SRD5A2* was significantly higher (*P* < 0.05) in the HFD group than in the NFD group on days 40 and 60 (Figure 7C).

## Discussion

China is the world’s largest producer of aquacultured tilapia (Malik et al., 2018). However, tilapia is susceptible to hepatic steatosis as a result of dietary imbalances in modern culture systems (Ng and Romano, 2013; Huang et al., 2016, 2018). The mechanism underlying the development of fatty liver in tilapia is still not fully understood. Our results confirmed that a HFD (18.5% lipids) quickly and effectively induced the formation of fatty liver in GIFT juveniles. Our results also suggested that the dysregulation of hepatic miRNAs (miR-122, miR-29a, miR-145-5p, and miR-34a) may disturb lipid homeostasis in GIFT juveniles by affecting the expression of their target genes in lipid metabolism.

Hepatic steatosis is strongly associated with abnormal lipid metabolism, which is mainly reflected as an imbalance between hepatic lipid inputs and outputs (Du et al., 2006; Lu et al., 2013b; Wang et al., 2015). When excessive lipids accumulate in the liver, the relevant pathways are activated and lipoproteins deliver lipids to the peripheral tissues (Lu et al., 2013b). Blood serves as an important metabolic transport system to regulate lipid metabolism. In this study, we found that serum TG, TC, LDL-C, and insulin levels were increased in GIFT fed a HFD, suggesting that endogenous metabolic transport was activated in these GIFT. An increased level of serum insulin would promote glucose uptake by the liver and consequently contribute to better glycemic control (Qiang et al., 2016). Similar results have been reported for blunt snout bream (Zhang et al., 2018). In fish, AST and ALT are two important transaminases that are mainly found in hepatocytes. Their activities in the serum may increase when the liver is damaged or inflamed (Sheikhzadeh et al., 2012). Together with the liver histological characteristics and hepatic lipid index assay results, the higher serum ALT activity observed in the HFD group suggested that excessive fat deposition in the liver not only altered hepatocytes, but also led to liver damage in GIFT.

Du et al. (2006) reported that dietary fatty acids strongly affect the fatty acid composition in fish tissues. In this study, we used soybean oil, which is rich in linoleic acid (C18:2n-6) and α-linolenic acid (C18:3n-3), as the oil source. This may have led to the high proportion of hepatic C18:2n-6 and C18:3n-3 in the HFD-fed GIFT. Linoleic and linolenic acids are considered as essential fatty acids (EFAs) for most animals, including fish (Holman, 1986). Fish can convert C18:2n-6 and C18:3n-3 into longer-chain, more unsaturated fatty acids (Tocher et al., 2001). In this study, the higher ratio of hepatic C20:2n-6 and C20:5n-3 in HFD-fed GIFT suggested that these fish may have been able to convert C18 EFAs into longer-chain n-3 and n-6 PUFA. We also found that the HFD led to an increase in the proportion of hepatic PUFA. A study on grass carp (Wang et al., 2016) suggested that fatty acid metabolic disorders may occur when fish are fed a HFD, because this leads to higher PUFA contents in the liver and possibly to the formation of fatty liver.

Excessive fat deposition in fish tissues can induce oxidative stress and impair fish health (Chaiyapechara et al., 2003; Zhang et al., 2018). The product of lipid peroxidation is MDA, and so the MDA level can indirectly reflect the degree of oxidative damage in fish tissues (Jiang et al., 2016). In fish, SOD, CAT, and GSH-Px are three important antioxidant enzymes; SOD can convert superoxide radicals into hydrogen peroxide, which can be further eliminated by GSH-Px and CAT (Jiang et al., 2016). In this study, hepatic GSH-Px and CAT activities were increased by day 20 in HFD group, indicating that short-term high-fat feeding (20 days) may activate the antioxidant system to remove excess oxygen free radicals. However, 60 days of high-fat rearing led to decreased hepatic SOD, CAT, and GSH-Px activities and an increased MDA content, consistent with the findings of studies on Chinese sucker (*Myxocyprinus asiaticus*) (Wang et al., 2010) and Japanese pufferfish (*Takifugu rubripes*) (Sun Y. et al., 2013). This suggested that excessive fat deposition in the liver may have led to a hepatic metabolic disorder, which attenuated hepatic antioxidant defenses and increased oxidative stress.

To investigate the possible mechanism of HFD-induced fatty liver in GIFT juveniles, we determined the expression levels of several miRNAs (miR-122, miR-29a, miR-145-5p, and miR-34a) and their predicted lipid metabolism-related target genes (*SCD*, *ELOVL6*, and *SRD5A2*) on days 20, 40, and 60. miR-122 is a liver-specific miRNA that was shown to account for almost 60% of total miRNAs in GIFT liver (Tao et al., 2017). The large amount of miR-122 in GIFT liver suggests that it plays a crucial role in regulating liver function. An increased expression level of miR-122 was linked with hepatic steatosis in zebra fish (Her et al., 2011). miR-29a is one of the mature forms of the miR-29 family and it is highly conserved in most species (Liston et al., 2012). Yang et al. (2014) reported that miR-29a was activated in C57BL/6 mice fed a HFD and the SFA palmitate, and that it functionally targeted the 3′-UTR of insulin receptor substrate-1 to regulate insulin signaling. We obtained a similar result, in that a HFD induced the expression of hepatic miR-122 and miR-29a in juvenile GIFT. SCD is a rate-limiting enzyme in the synthesis of MUFA from SFA (Heinemann and Ozols, 2003). An SCD1 deficiency in mouse led to hyperphagy, but the mice were lean and protected from HFD-induced obesity (Miyazaki et al., 2009). Ntambi (1999) reported that MUFA are more likely than SFA to combine with acyl-CoA cholesterol and triglycerides to produce TG and TC, respectively. In this study, *SCD* expression was suppressed in the HFD group. The results suggested that miR-122 together with miR-29a may be involved in reducing the synthesis of MUFA through attenuating the expression levels of *SCD*. This may contribute to the control of fat synthesis (especially TG and TC).

Wang et al. (2017) reported that miR-145 is a key regulator of lipogenesis in goat mammary cells. Overexpression of miR-145 activated the expression of fat synthesis-related genes at the transcript level, resulting in greater fat accumulation. The expression levels of hepatic miR-145 decreased in blunt snout bream fed a HFD (Zhang et al., 2014). However, in this study, miR-145-5p expression was increased in the liver of HFD-fed GIFT on day 60. We speculated that, different from other fish species, GIFT may have some other adaptation strategies to a HFD. ELOVL6, the only elongase involved in *de novo* lipogenesis, catalyzes the rate-limiting step in 18-C fatty acid synthesis (Moon et al., 2001). A deficiency of ELOVL6 was shown to alter the ratio of hepatic fatty acids in mice (Matsuzaka et al., 2007). Inhibition of this elongase could be a new therapeutic approach for ameliorating diabetes (Matsuzaka et al., 2007; Sun H. et al., 2013). Several studies have reported that fish can change their metabolic strategy (to decrease lipogenesis and increase β-oxidation of fatty acids) to adapt to a high fat intake (He A.Y. et al., 2015; Li et al., 2016). In this study, the proportion of C18:2n-6 was almost 3.5-fold higher in the HFD group than in the NFD. We speculated that in juvenile GIFT fed a HFD, to maintain fatty acid homeostasis, the levels of miR-122, miR-29a, and miR-145-5p increased and the transcript levels of *SCD* and *ELOVL6* decreased to reduce the conversion of fatty acids into C18:2n-6.

In mammals, miR-34a is thought to be involved in non-alcoholic fatty liver disease (Ding et al., 2015). Moreover, miR-34a^-/-^ mice were shown to be susceptible to HFD-induced obesity (Lavery et al., 2016). Nasiri et al. (2015) reported that SRD5A2 is involved in regulating lipid homeostasis in human hepatocytes. Overexpression of *SRD5A2* was shown to inhibit the effects of cortisol and thereby suppress lipogenesis. In this study, a HFD downregulated miR-34a expression but upregulated *SRD5A2* in the liver of GIFT. This result suggested that the interaction between miR-34a and *SRD5A2* mRNA may play an important role in lipid metabolism in GIFT fed a HFD.

## Conclusion

The results of our study indicated that a HFD can cause excess fat deposition in the liver of GIFT juveniles. The excess fat deposition in the liver altered serum parameters, hepatic fatty acid composition, and antioxidant enzyme activities. This is the first report of differential expression patterns of four miRNAs (miR-122, miR-29a, miR-145-5p, and miR-34a) and their potential target genes (*SCD*, *ELOVL6*, and *SRD5A2*) in the liver of GIFT during 60 days of growth on a HFD. Our results suggested that these four miRNAs may be involved in lipid metabolism by post-transcriptionally regulating the expression levels of *SCD*, *ELOVL6*, and *SRD5A2*. Further research including western blotting and luciferase reporter assays is required to validate the functions of miR-122-*SCD*, miR-29a-*SCD*, miR-145-5p-*ELOVL6*, and miR-34a-*SRD5A2* pairs in the lipid metabolism of GIFT.

## Author Contributions

G-JY and PX conceived and designed the experiments. Y-FT, JQ, J-WB, D-JC, and H-JZ performed the experiments. Y-FT and JQ analyzed the data and wrote the manuscript. All authors read and approved the final version of the manuscript.

## Conflict of Interest Statement

The authors declare that the research was conducted in the absence of any commercial or financial relationships that could be construed as a potential conflict of interest.

## Footnotes

1 http://www.microrna.org/

2 http://www.ncbi.nlm.nih.gov/genome/?term=Oreochromis%20niloticus

3 http://www.geneontology.org/

## Supplementary Material

Table S1 Output of the ANOVA analysis.
