# Supplementary material for: Changes in Physiological Parameters, Lipid Metabolism, and Expression of MicroRNAs in Genetically Improved Farmed Tilapia (Oreochromis niloticus) With Fatty Liver Induced by a High-Fat Diet
Source: Front Physiol. 2018 Oct 30;9:1521. doi: 10.3389/fphys.2018.01521 (PMC6218568; doi:10.3389/fphys.2018.01521)
Supplement: Table S1 — Output of the ANOVA analysis. [file Table_1.DOCX]

Results of one-way ANOVA test of serum TG

|  | df (between group) | df (within group) | F | Sig. |
| --- | --- | --- | --- | --- |
| Values in HFD group among different sampling points | 2 | 24 | 11.593 | * |
| Values in NFD group among different sampling points | 2 | 24 | 2.788 | NS |

Note: *: *p* < 0.05, NS: no significant difference.

Results of post-hoc Duncan’s multiple range test of serum TG in GIFT fed HFD

|  | N | Subset for alpha = 0.05 | |
| --- | --- | --- | --- |
|  |  | 1 | 2 |
| 20d | 9 | 0.927 |  |
| 40d | 9 | 1.154 |  |
| 60d | 9 |  | 2.607 |

Results of independent-sample t test of serum TG

|  | t | df | Sig.(two tail) |
| --- | --- | --- | --- |
| Values between the HFD and NFD groups at 20 d | -0.326 | 16 | NS |
| Values between the HFD and NFD groups at 40 d | -0.341 | 16 | NS |
| Values between the HFD and NFD groups at 60 d | 2.390 | 16 | * |

Note: *: *p* < 0.05, NS: no significant difference.

Results of one-way ANOVA test of serum TC

|  | df (between group) | df (within group) | F | Sig. |
| --- | --- | --- | --- | --- |
| Values in HFD group among different sampling points | 2 | 24 | 4.938 | * |
| Values in NFD group among different sampling points | 2 | 24 | 10.932 | * |

Note: *: *p* < 0.05

Results of post-hoc Duncan’s multiple range test of serum TC in GIFT fed HFD

|  | N | Subset for alpha = 0.05 | |
| --- | --- | --- | --- |
|  |  | 1 | 2 |
| 20d | 9 | 4.912 |  |
| 40d | 9 |  | 6.594 |
| 60d | 9 |  | 6.615 |

Results of post-hoc Duncan’s multiple range test of serum TC in GIFT fed NFD

|  | N | Subset for alpha = 0.05 | |
| --- | --- | --- | --- |
|  |  | 1 | 2 |
| 20d | 9 | 2.488 |  |
| 40d | 9 |  | 3.304 |
| 60d | 9 |  | 3.932 |

Results of independent-sample t test of serum TC

|  | t | df | Sig.(two tail) |
| --- | --- | --- | --- |
| Values between the HFD and NFD groups at 20 d | 7.526 | 16 | * |
| Values between the HFD and NFD groups at 40 d | 5.857 | 16 | * |
| Values between the HFD and NFD groups at 60 d | 4.863 | 16 | * |

Note: *: *p* < 0.05

Results of one-way ANOVA test of serum LDL-C

|  | df (between group) | df (within group) | F | Sig. |
| --- | --- | --- | --- | --- |
| Values in HFD group among different sampling points | 2 | 24 | 4.073 | * |
| Values in NFD group among different sampling points | 2 | 24 | 0.324 | NS |

Note: *: *p* < 0.05, NS: no significant difference.

Results of post-hoc Duncan’s multiple range test of serum LDL-C in GIFT fed HFD

|  | N | Subset for alpha = 0.05 | |
| --- | --- | --- | --- |
|  |  | 1 | 2 |
| 20d | 9 | 0.727 |  |
| 40d | 9 |  | 0.993 |
| 60d | 9 |  | 1.074 |

Results of independent-sample t test of serum LDL-C

|  | t | df | Sig.(two tail) |
| --- | --- | --- | --- |
| Values between the HFD and NFD groups at 20 d | -0.413 | 16 | NS |
| Values between the HFD and NFD groups at 40 d | 2.749 | 16 | * |
| Values between the HFD and NFD groups at 60 d | 2.826 | 16 | * |

Note: *: *p* < 0.05, NS: no significant difference.

Results of one-way ANOVA test of serum HDL-C

|  | df (between group) | df (within group) | F | Sig. |
| --- | --- | --- | --- | --- |
| Values in HFD group among different sampling points | 2 | 24 | 1.026 | NS |
| Values in NFD group among different sampling points | 2 | 24 | 0.246 | NS |

Note: NS: no significant difference

Results of independent-sample t test of serum HDL-C

|  | t | df | Sig.(two tail) |
| --- | --- | --- | --- |
| Values between the HFD and NFD groups at 20 d | 0.215 | 16 | NS |
| Values between the HFD and NFD groups at 40 d | 0.610 | 16 | NS |
| Values between the HFD and NFD groups at 60 d | -0.357 | 16 | NS |

Note: NS: no significant difference

Results of one-way ANOVA test of serum glucose

|  | df (between group) | df (within group) | F | Sig. |
| --- | --- | --- | --- | --- |
| Values in HFD group among different sampling points | 2 | 24 | 2.716 | NS |
| Values in NFD group among different sampling points | 2 | 24 | 1.846 | NS |

Note: NS: no significant difference.

Results of independent-sample t test of serum glucose

|  | t | df | Sig.(two tail) |
| --- | --- | --- | --- |
| Values between the HFD and NFD groups at 20 d | -0.866 | 16 | NS |
| Values between the HFD and NFD groups at 40 d | 1.596 | 16 | NS |
| Values between the HFD and NFD groups at 60 d | 2.100 | 16 | NS |

Note: NS: no significant difference.

Results of one-way ANOVA test of serum insulin

|  | df (between group) | df (within group) | F | Sig. |
| --- | --- | --- | --- | --- |
| Values in HFD group among different sampling points | 2 | 24 | 3.460 | * |
| Values in NFD group among different sampling points | 2 | 24 | 0.677 | NS |

Note: *: *p* < 0.05, NS: no significant difference.

Results of post-hoc Duncan’s multiple range test of serum insulin in GIFT fed HFD

|  | N | Subset for alpha = 0.05 | |
| --- | --- | --- | --- |
|  |  | 1 | 2 |
| 20d | 9 | 1.915 |  |
| 40d | 9 | 2.435 | 2.435 |
| 60d | 9 |  | 2.721 |

Results of independent-sample t test of serum insulin

|  | t | df | Sig.(two tail) |
| --- | --- | --- | --- |
| Values between the HFD and NFD groups at 20 d | 0.858 | 16 | NS |
| Values between the HFD and NFD groups at 40 d | 2.826 | 16 | * |
| Values between the HFD and NFD groups at 60 d | 2.408 | 16 | * |

Note: *: *p* < 0.05, NS: no significant difference.

Results of one-way ANOVA test of serum ALT

|  | df (between group) | df (within group) | F | Sig. |
| --- | --- | --- | --- | --- |
| Values in HFD group among different sampling points | 2 | 24 | 5.748 | * |
| Values in NFD group among different sampling points | 2 | 24 | 2.285 | NS |

Note: *: *p* < 0.05, NS: no significant difference.

Results of post-hoc Duncan’s multiple range test of serum ALT in GIFT fed HFD

|  | N | Subset for alpha = 0.05 | |
| --- | --- | --- | --- |
|  |  | 1 | 2 |
| 20d | 9 | 15.489 |  |
| 40d | 9 | 18.994 |  |
| 60d | 9 |  | 26.825 |

Results of independent-sample t test of serum ALT

|  | t | df | Sig.(two tail) |
| --- | --- | --- | --- |
| Values between the HFD and NFD groups at 20 d | 3.560 | 16 | * |
| Values between the HFD and NFD groups at 40 d | 4.206 | 16 | * |
| Values between the HFD and NFD groups at 60 d | 4.147 | 16 | * |

Note: *: *p* < 0.05

Results of one-way ANOVA test of serum AST

|  | df (between group) | df (within group) | F | Sig. |
| --- | --- | --- | --- | --- |
| Values in HFD group among different sampling points | 2 | 24 | 0.473 | NS |
| Values in NFD group among different sampling points | 2 | 24 | 0.142 | NS |

Note: NS: no significant difference.

Results of independent-sample t test of serum AST

|  | t | df | Sig.(two tail) |
| --- | --- | --- | --- |
| Values between the HFD and NFD groups at 20 d | 0.626 | 16 | NS |
| Values between the HFD and NFD groups at 40 d | 0.183 | 16 | NS |
| Values between the HFD and NFD groups at 60 d | 1.750 | 16 | NS |

Note: NS: no significant difference.

Results of one-way ANOVA test of hepatic TG

|  | df (between group) | df (within group) | F | Sig. |
| --- | --- | --- | --- | --- |
| Values in HFD group among different sampling points | 2 | 24 | 4.093 | * |
| Values in NFD group among different sampling points | 2 | 24 | 2.481 | NS |

Note: *: *p* < 0.05, NS: no significant difference.

Results of post-hoc Duncan’s multiple range test of hepatic TG in GIFT fed HFD

|  | N | Subset for alpha = 0.05 | |
| --- | --- | --- | --- |
|  |  | 1 | 2 |
| 20d | 9 | 9.328 |  |
| 60d | 9 |  | 16.069 |
| 40d | 9 |  | 16.263 |

Results of independent-sample t test of hepatic TG

|  | t | df | Sig.(two tail) |
| --- | --- | --- | --- |
| Values between the HFD and NFD groups at 20 d | 3.622 | 16 | * |
| Values between the HFD and NFD groups at 40 d | 5.643 | 16 | * |
| Values between the HFD and NFD groups at 60 d | 2.836 | 16 | * |

Note: *: *p* < 0.05

Results of one-way ANOVA test of hepatic TC

|  | df (between group) | df (within group) | F | Sig. |
| --- | --- | --- | --- | --- |
| Values in HFD group among different sampling points | 2 | 24 | 1.056 | NS |
| Values in NFD group among different sampling points | 2 | 24 | 2.271 | NS |

Note: NS: no significant difference.

Results of independent-sample t test of hepatic TC

|  | t | df | Sig.(two tail) |
| --- | --- | --- | --- |
| Values between the HFD and NFD groups at 20 d | 3.084 | 16 | * |
| Values between the HFD and NFD groups at 40 d | 2.268 | 16 | * |
| Values between the HFD and NFD groups at 60 d | 3.548 | 16 | * |

Note: *: *p* < 0.05

Results of one-way ANOVA test of hepatic SOD

|  | df (between group) | df (within group) | F | Sig. |
| --- | --- | --- | --- | --- |
| Values in HFD group among different sampling points | 2 | 24 | 32.475 | * |
| Values in NFD group among different sampling points | 2 | 24 | 0.350 | NS |

Note: *: *p* < 0.05, NS: no significant difference.

Results of post-hoc Duncan’s multiple range test of hepatic SOD in GIFT fed HFD

|  | N | Subset for alpha = 0.05 | |
| --- | --- | --- | --- |
|  |  | 1 | 2 |
| 60d | 9 | 27.375 |  |
| 40d | 9 |  | 42.959 |
| 20d | 9 |  | 46.119 |

Results of independent-sample t test of hepatic SOD

|  | t | df | Sig.(two tail) |
| --- | --- | --- | --- |
| Values between the HFD and NFD groups at 20 d | 0.341 | 16 | NS |
| Values between the HFD and NFD groups at 40 d | -0.790 | 16 | NS |
| Values between the HFD and NFD groups at 60 d | -7.220 | 16 | * |

Note: *: *p* < 0.05, NS: no significant difference.

Results of one-way ANOVA test of hepatic CAT

|  | df (between group) | df (within group) | F | Sig. |
| --- | --- | --- | --- | --- |
| Values in HFD group among different sampling points | 2 | 24 | 18.833 | * |
| Values in NFD group among different sampling points | 2 | 24 | 0.392 | NS |

Note: *: *p* < 0.05, NS: no significant difference.

Results of post-hoc Duncan’s multiple range test of hepatic CAT in GIFT fed HFD

|  | N | Subset for alpha = 0.05 | | |
| --- | --- | --- | --- | --- |
|  |  | 1 | 2 | 3 |
| 60d | 9 | 7.196 |  |  |
| 40d | 9 |  | 12.365 |  |
| 20d | 9 |  |  | 17.265 |

Results of independent-sample t test of hepatic CAT

|  | t | df | Sig.(two tail) |
| --- | --- | --- | --- |
| Values between the HFD and NFD groups at 20 d | 2.861 | 16 | * |
| Values between the HFD and NFD groups at 40 d | 0.420 | 16 | NS |
| Values between the HFD and NFD groups at 60 d | -2.758 | 16 | * |

Note: *: *p* < 0.05, NS: no significant difference.

Results of one-way ANOVA test of hepatic GSH-Px

|  | df (between group) | df (within group) | F | Sig. |
| --- | --- | --- | --- | --- |
| Values in HFD group among different sampling points | 2 | 24 | 30.329 | * |
| Values in NFD group among different sampling points | 2 | 24 | 0.790 | NS |

Note: *: *p* < 0.05, NS: no significant difference.

Results of post-hoc Duncan’s multiple range test of hepatic GSH-Px in GIFT fed HFD

|  | N | Subset for alpha = 0.05 | | |
| --- | --- | --- | --- | --- |
|  |  | 1 | 2 | 3 |
| 60d | 9 | 100.658 |  |  |
| 40d | 9 |  | 257.867 |  |
| 20d | 9 |  |  | 430.787 |

Results of independent-sample t test of hepatic GSH-Px

|  | t | df | Sig.(two tail) |
| --- | --- | --- | --- |
| Values between the HFD and NFD groups at 20 d | 2.465 | 16 | * |
| Values between the HFD and NFD groups at 40 d | -1.686 | 16 | NS |
| Values between the HFD and NFD groups at 60 d | -4.276 | 16 | * |

Note: *: *p* < 0.05, NS: no significant difference.

Results of one-way ANOVA test of hepatic MDA

|  | df (between group) | df (within group) | F | Sig. |
| --- | --- | --- | --- | --- |
| Values in HFD group among different sampling points | 2 | 24 | 18.009 | * |
| Values in NFD group among different sampling points | 2 | 24 | 2.403 | NS |

Note: *: *p* < 0.05, NS: no significant difference.

Results of post-hoc Duncan’s multiple range test of hepatic MDA in GIFT fed HFD

|  | N | Subset for alpha = 0.05 | |
| --- | --- | --- | --- |
|  |  | 1 | 2 |
| 20d | 9 | 1.489 |  |
| 40d | 9 | 2.848 |  |
| 60d | 9 |  | 5.758 |

Results of independent-sample t test of hepatic MDA

|  | t | df | Sig.(two tail) |
| --- | --- | --- | --- |
| Values between the HFD and NFD groups at 20 d | 1.697 | 16 | NS |
| Values between the HFD and NFD groups at 40 d | 5.107 | 16 | * |
| Values between the HFD and NFD groups at 60 d | 5.825 | 16 | * |

Note: *: *p* < 0.05, NS: no significant difference.

Results of one-way ANOVA test of hepatic miR-122

|  | df (between group) | df (within group) | F | Sig. |
| --- | --- | --- | --- | --- |
| Values in HFD group among different sampling points | 2 | 24 | 7.767 | * |
| Values in NFD group among different sampling points | 2 | 24 | 0.501 | NS |

Note: *: *p* < 0.05, NS: no significant difference.

Results of post-hoc Duncan’s multiple range test of hepatic miR-122 in GIFT fed HFD

|  | N | Subset for alpha = 0.05 | |
| --- | --- | --- | --- |
|  |  | 1 | 2 |
| 60d | 9 | 1.033 |  |
| 40d | 9 |  | 1.563 |
| 20d | 9 |  | 1.691 |

Results of independent-sample t test of hepatic miR-122

|  | t | df | Sig.(two tail) |
| --- | --- | --- | --- |
| Values between the HFD and NFD groups at 20 d | 3.738 | 16 | * |
| Values between the HFD and NFD groups at 40 d | 3.198 | 16 | * |
| Values between the HFD and NFD groups at 60 d | 0.517 | 16 | NS |

Note: *: *p* < 0.05, NS: no significant difference.

Results of one-way ANOVA test of hepatic miR-29a

|  | df (between group) | df (within group) | F | Sig. |
| --- | --- | --- | --- | --- |
| Values in HFD group among different sampling points | 2 | 24 | 12.366 | * |
| Values in NFD group among different sampling points | 2 | 24 | 7.379 | * |

Note: *: *p* < 0.05

Results of post-hoc Duncan’s multiple range test of hepatic miR-29a in GIFT fed HFD

|  | N | Subset for alpha = 0.05 | | |
| --- | --- | --- | --- | --- |
|  |  | 1 | 2 | 3 |
| 20d | 9 | 0.775 |  |  |
| 40d | 9 |  | 1.413 |  |
| 60d | 9 |  |  | 2.125 |

Results of post-hoc Duncan’s multiple range test of hepatic miR-29a in GIFT fed NFD

|  | N | Subset for alpha = 0.05 | |
| --- | --- | --- | --- |
|  |  | 1 | 2 |
| 20d | 9 | 0.841 |  |
| 40d | 9 | 0.903 |  |
| 60d | 9 |  | 1.251 |

Results of independent-sample t test of hepatic miR-29a

|  | t | df | Sig.(two tail) |
| --- | --- | --- | --- |
| Values between the HFD and NFD groups at 20 d | -0.853 | 16 | NS |
| Values between the HFD and NFD groups at 40 d | 3.060 | 16 | * |
| Values between the HFD and NFD groups at 60 d | 2.812 | 16 | * |

Note: *: *p* < 0.05, NS: no significant difference.

Results of one-way ANOVA test of hepatic miR-145-5p

|  | df (between group) | df (within group) | F | Sig. |
| --- | --- | --- | --- | --- |
| Values in HFD group among different sampling points | 2 | 24 | 4.721 | * |
| Values in NFD group among different sampling points | 2 | 24 | 0.633 | NS |

Note: *: *p* < 0.05, NS: no significant difference.

Results of post-hoc Duncan’s multiple range test of hepatic miR-145-5p in GIFT fed HFD

|  | N | Subset for alpha = 0.05 | |
| --- | --- | --- | --- |
|  |  | 1 | 2 |
| 20d | 9 | 0.830 |  |
| 40d | 9 |  | 1.261 |
| 60d | 9 |  | 1.440 |

Results of independent-sample t test of hepatic miR-145-5p

|  | t | df | Sig.(two tail) |
| --- | --- | --- | --- |
| Values between the HFD and NFD groups at 20 d | -1.127 | 16 | NS |
| Values between the HFD and NFD groups at 40 d | 1.138 | 16 | NS |
| Values between the HFD and NFD groups at 60 d | 2.814 | 16 | * |

Note: *: *p* < 0.05, NS: no significant difference.

Results of one-way ANOVA test of hepatic miR-34a

|  | df (between group) | df (within group) | F | Sig. |
| --- | --- | --- | --- | --- |
| Values in HFD group among different sampling points | 2 | 24 | 11.451 | * |
| Values in NFD group among different sampling points | 2 | 24 | 9.809 | * |

Note: *: *p* < 0.05

Results of post-hoc Duncan’s multiple range test of hepatic miR-34a in GIFT fed HFD

|  | N | Subset for alpha = 0.05 | |
| --- | --- | --- | --- |
|  |  | 1 | 2 |
| 60d | 9 | 0.124 |  |
| 40d | 9 | 0.297 |  |
| 20d | 9 |  | 0.616 |

Results of post-hoc Duncan’s multiple range test of hepatic miR-34a in GIFT fed NFD

|  | N | Subset for alpha = 0.05 | |
| --- | --- | --- | --- |
|  |  | 1 | 2 |
| 60d | 9 | 0.284 |  |
| 20d | 9 |  | 0.721 |
| 40d | 9 |  | 0.939 |

Results of independent-sample t test of hepatic miR-34a

|  | t | df | Sig.(two tail) |
| --- | --- | --- | --- |
| Values between the HFD and NFD groups at 20 d | -0.702 | 16 | NS |
| Values between the HFD and NFD groups at 40 d | -4.036 | 16 | * |
| Values between the HFD and NFD groups at 60 d | -3.038 | 16 | * |

Note: *: *p* < 0.05, NS: no significant difference.

Results of one-way ANOVA test of hepatic SCD

|  | df (between group) | df (within group) | F | Sig. |
| --- | --- | --- | --- | --- |
| Values in HFD group among different sampling points | 2 | 24 | 6.083 | * |
| Values in NFD group among different sampling points | 2 | 24 | 3.769 | * |

Note: *: *p* < 0.05

Results of post-hoc Duncan’s multiple range test of hepatic SCD in GIFT fed HFD

|  | N | Subset for alpha = 0.05 | |
| --- | --- | --- | --- |
|  |  | 1 | 2 |
| 40d | 9 | 0.009 |  |
| 20d | 9 | 0.023 |  |
| 60d | 9 |  | 0.059 |

Results of post-hoc Duncan’s multiple range test of hepatic SCD in GIFT fed NFD

|  | N | Subset for alpha = 0.05 | |
| --- | --- | --- | --- |
|  |  | 1 | 2 |
| 40d | 9 | 0.937 |  |
| 60d | 9 | 0.992 |  |
| 20d | 9 |  | 1.420 |

Results of independent-sample t test of hepatic SCD

|  | t | df | Sig.(two tail) |
| --- | --- | --- | --- |
| Values between the HFD and NFD groups at 20 d | -8.780 | 16 | * |
| Values between the HFD and NFD groups at 40 d | -9.699 | 16 | * |
| Values between the HFD and NFD groups at 60 d | -6.368 | 16 | * |

Note: *: *p* < 0.05

Results of one-way ANOVA test of hepatic ELOVL6

|  | df (between group) | df (within group) | F | Sig. |
| --- | --- | --- | --- | --- |
| Values in HFD group among different sampling points | 2 | 24 | 4.857 | * |
| Values in NFD group among different sampling points | 2 | 24 | 8.067 | * |

Note: *: *p* < 0.05

Results of post-hoc Duncan’s multiple range test of hepatic ELOVL6 in GIFT fed HFD

|  | N | Subset for alpha = 0.05 | |
| --- | --- | --- | --- |
|  |  | 1 | 2 |
| 20d | 9 | 0.605 |  |
| 40d | 9 | 0.788 |  |
| 60d | 9 |  | 1.327 |

Results of post-hoc Duncan’s multiple range test of hepatic ELOVL6 in GIFT fed NFD

|  | N | Subset for alpha = 0.05 | |
| --- | --- | --- | --- |
|  |  | 1 | 2 |
| 20d | 9 | 0.954 |  |
| 40d | 9 | 1.312 |  |
| 60d | 9 |  | 2.309 |

Results of independent-sample t test of hepatic ELOVL6

|  | t | df | Sig.(two tail) |
| --- | --- | --- | --- |
| Values between the HFD and NFD groups at 20 d | -1.327 | 16 | NS |
| Values between the HFD and NFD groups at 40 d | -2.406 | 16 | * |
| Values between the HFD and NFD groups at 60 d | -2.500 | 16 | * |

Note: *: *p* < 0.05, NS: no significant difference.

Results of one-way ANOVA test of hepatic SRD5A2

|  | df (between group) | df (within group) | F | Sig. |
| --- | --- | --- | --- | --- |
| Values in HFD group among different sampling points | 2 | 24 | 20.164 | * |
| Values in NFD group among different sampling points | 2 | 24 | 4.353 | * |

Note: *: *p* < 0.05

Results of post-hoc Duncan’s multiple range test of hepatic SRD5A2 in GIFT fed HFD

|  | N | Subset for alpha = 0.05 | | |
| --- | --- | --- | --- | --- |
|  |  | 1 | 2 | 3 |
| 20d | 9 | 1.017 |  |  |
| 40d | 9 |  | 1.521 |  |
| 60d | 9 |  |  | 2.412 |

Results of post-hoc Duncan’s multiple range test of hepatic SRD5A2 in GIFT fed NFD

|  | N | Subset for alpha = 0.05 | |
| --- | --- | --- | --- |
|  |  | 1 | 2 |
| 20d | 9 | 0.930 |  |
| 40d | 9 | 1.043 |  |
| 60d | 9 |  | 1.419 |

Results of independent-sample t test of hepatic SRD5A2

|  | t | df | Sig.(two tail) |
| --- | --- | --- | --- |
| Values between the HFD and NFD groups at 20 d | 0.607 | 16 | NS |
| Values between the HFD and NFD groups at 40 d | 2.591 | 16 | * |
| Values between the HFD and NFD groups at 60 d | 3.896 | 16 | * |

Note: *: *p* < 0.05, NS: no significant difference.

Results of independent-sample t test of hepatic fatty acid composition

|  | t | | df | Sig.(two tail) |
| --- | --- | --- | --- | --- |
| C12:0 | | -6.668 | 16 | * |
| C14:0 | | -7.059 | 16 | * |
| C15:0 | | 6.798 | 16 | * |
| C16:0 | | -12.365 | 16 | * |
| C17:0 | | 8.815 | 16 | * |
| C18:0 | | -9.246 | 16 | * |
| C20:0 | | -1.286 | 16 | NS |
| C22:0 | | -1.395 | 16 | NS |
| ∑SFA | | -10.953 | 16 | * |
| C16:1 | | -20.838 | 16 | * |
| C18:1 | | -2.928 | 16 | * |
| C20:1 | | -8.476 | 16 | * |
| C22:1 | | 12.911 | 16 | * |
| ∑MUFA | | -3.767 | 16 | * |
| C18:2n-6 | | 13.563 | 16 | * |
| C18:3n-3 | | 12.429 | 16 | * |
| C18:3n-6 | | 6.134 | 16 | * |
| C20:2n-6 | | 7.639 | 16 | * |
| C20:3n-3 | | 1.465 | 16 | NS |
| C20:4n-6 | | -0.689 | 16 | NS |
| C20:5n-3 (EPA) | | 9.454 | 16 | * |
| C22:3 | | -1.204 | 16 | NS |
| C22:4n-6 | | -1.645 | 16 | NS |
| C22:5n-3 | | -1.009 | 16 | NS |
| C22:6n-3 (DHA) | | -2.011 | 16 | NS |
| ∑PUFA | | 8.763 | 16 | * |
| ∑n-3PUFA | | 0.436 | 16 | NS |
| ∑n-6PUFA | | 12.129 | 16 | * |

Note: *: *p* < 0.05, NS: no significant difference.
